# Supplementary material for: Enhancing the Treatment of Uncontrolled Inflammation through the Targeted Delivery of TPCA-1-Loaded Nanoparticles
Source: Pharmaceutics. 2023 Oct 9;15(10):2435. doi: 10.3390/pharmaceutics15102435 (PMC10609852; doi:10.3390/pharmaceutics15102435)
Supplement: Supplementary file 1 [file pharmaceutics-15-02435-s001.zip › pharmaceutics-2540022-supplementary.pdf]

Supplementary Materials: Treatment of Uncontrolled Inflammation through Targeted Delivery of TPCA-1-Loaded Nanoparticles

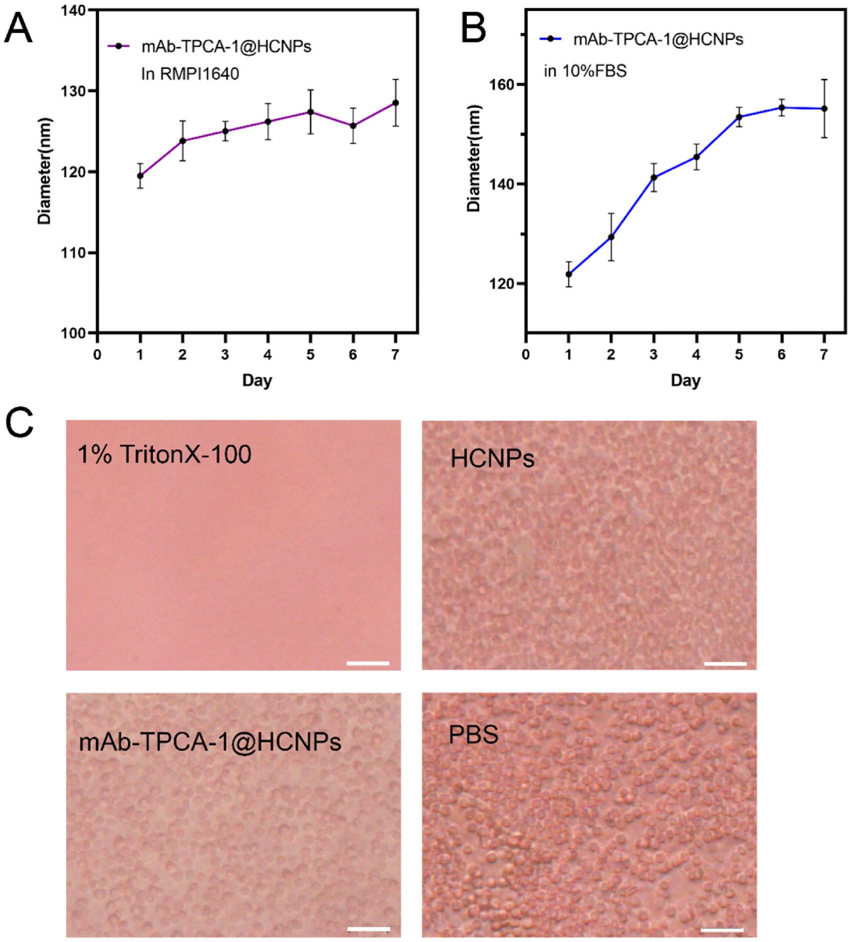

Figure S1. The size of mAb-TPCA-1@HCNPs in RPMI 1640 (A) and 10% FBS (B) during one week of storage at 4°C. (C) Representative microscopy images of erythrocytes with different treatments: 1% Triton X-100, HCNPs (500 µg/ml), mAb-TPCA-1@HCNPs (500 µg/ml) and PBS. The scale bar is 50 µm.

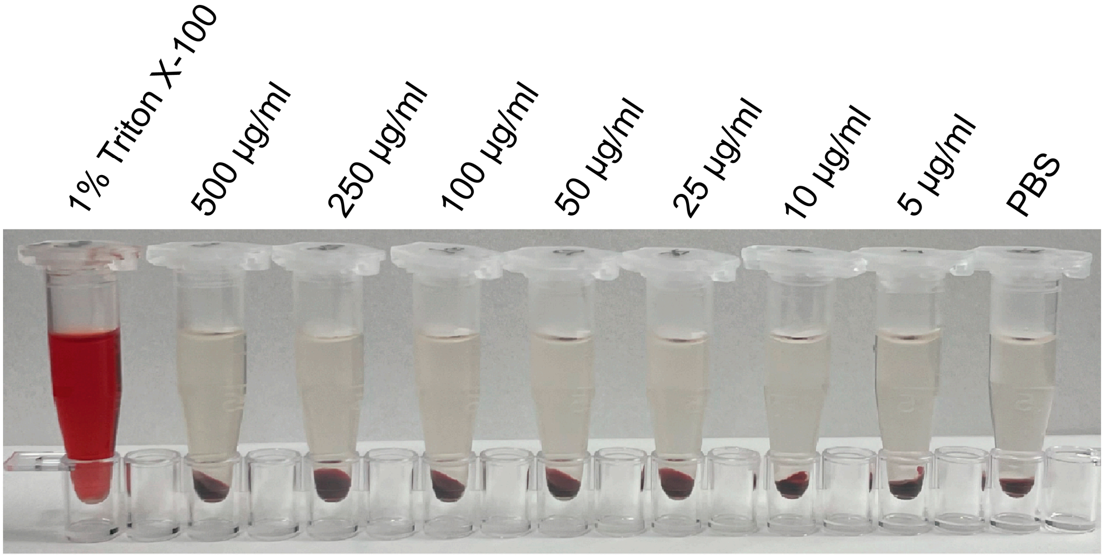

Figure S2. The photograph of Eppendorf tubes containing red blood cells treated with mAb-TPCA-1@HCNPs at different concentrations.

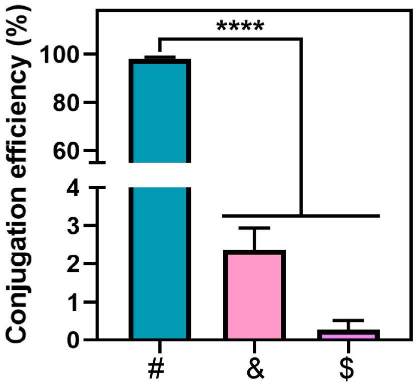

Figure S3. The quantitative analysis of the conjugation efficiency of mAbs to the TPCA-1-loaded NPs detected by nano-flow cytometry. Groups #, & and $ represent mAb-TPCA-1@HCNPs, TPCA-1@HCNPs and Blank NPs, respectively.

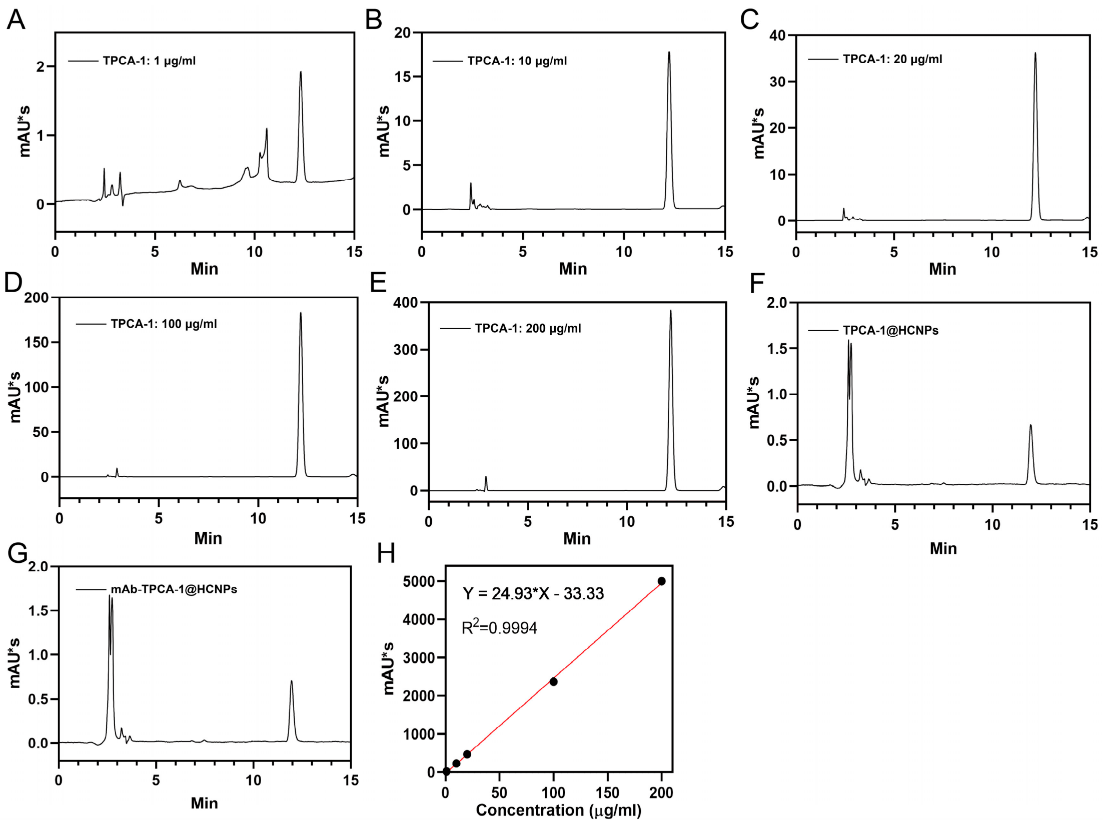

Figure S4. The HPLC result of free TPCA-1 at various concentration (A~E) and that released from TPCA-1@HCNPs (F) and mAb-TPCA-1@HCNPs (G) at 270 nm. The standard curve of TPCA-1 determined by HPLC (H).

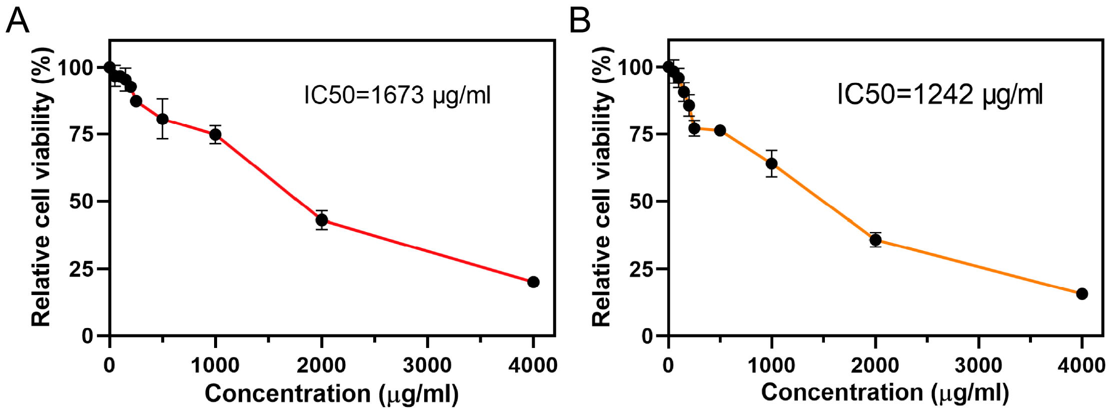

Figure S5. Cell cytotoxicity evaluation of HUVECs (A) and RAW264.7 cells (B) indicated by broken line graph, and the IC50 was 1673 μg/ml and 1242 μg/ml, respectively.

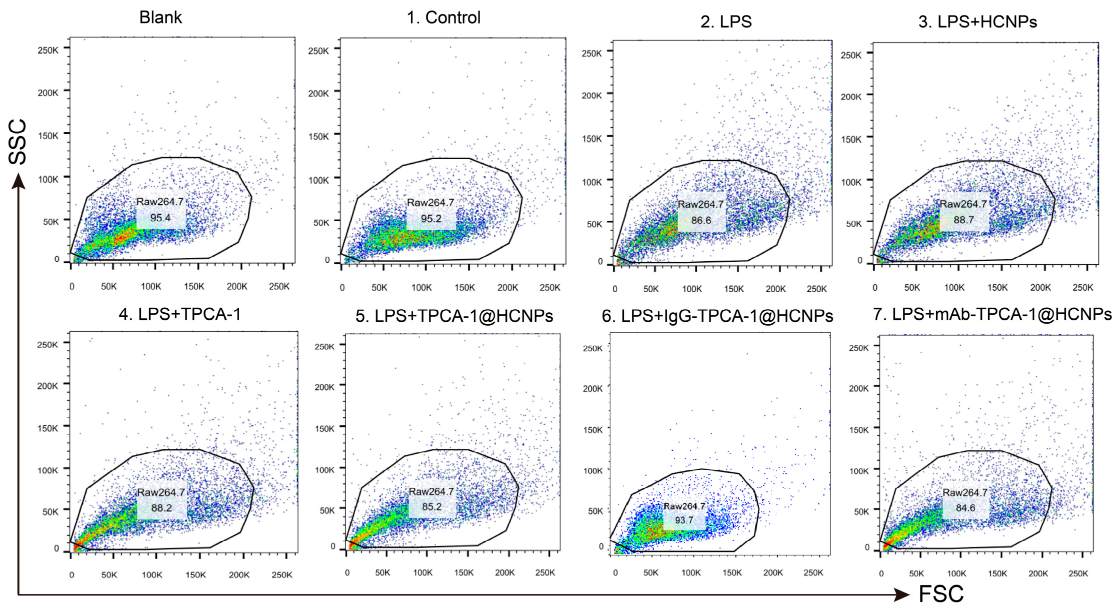

Figure S6. Gating strategy used for macrophage polarization experiment of RAW264.7 cells.

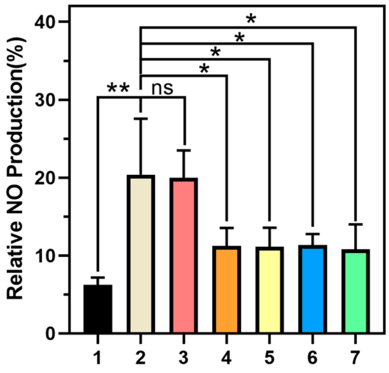

Figure S7. NO production of RAW264.7 cells treated with LPS (100 ng/mL), w/or w/o TPCA-1, HCNPs, TPCA-1@HCNPs or mAb-TPCA-1@HCNPs at 0.02 µg/ml TPCA-1 for 24 h.

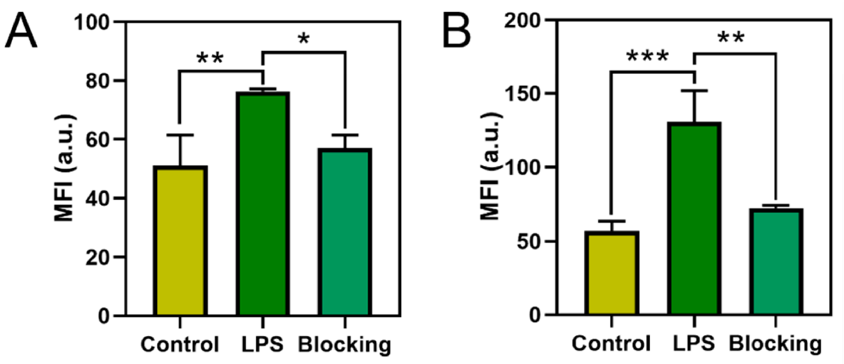

Figure S8. Fluorescence quantification of mAb-C6@HCNPs per unit area on macrophages (A) and endothelial cells (B).

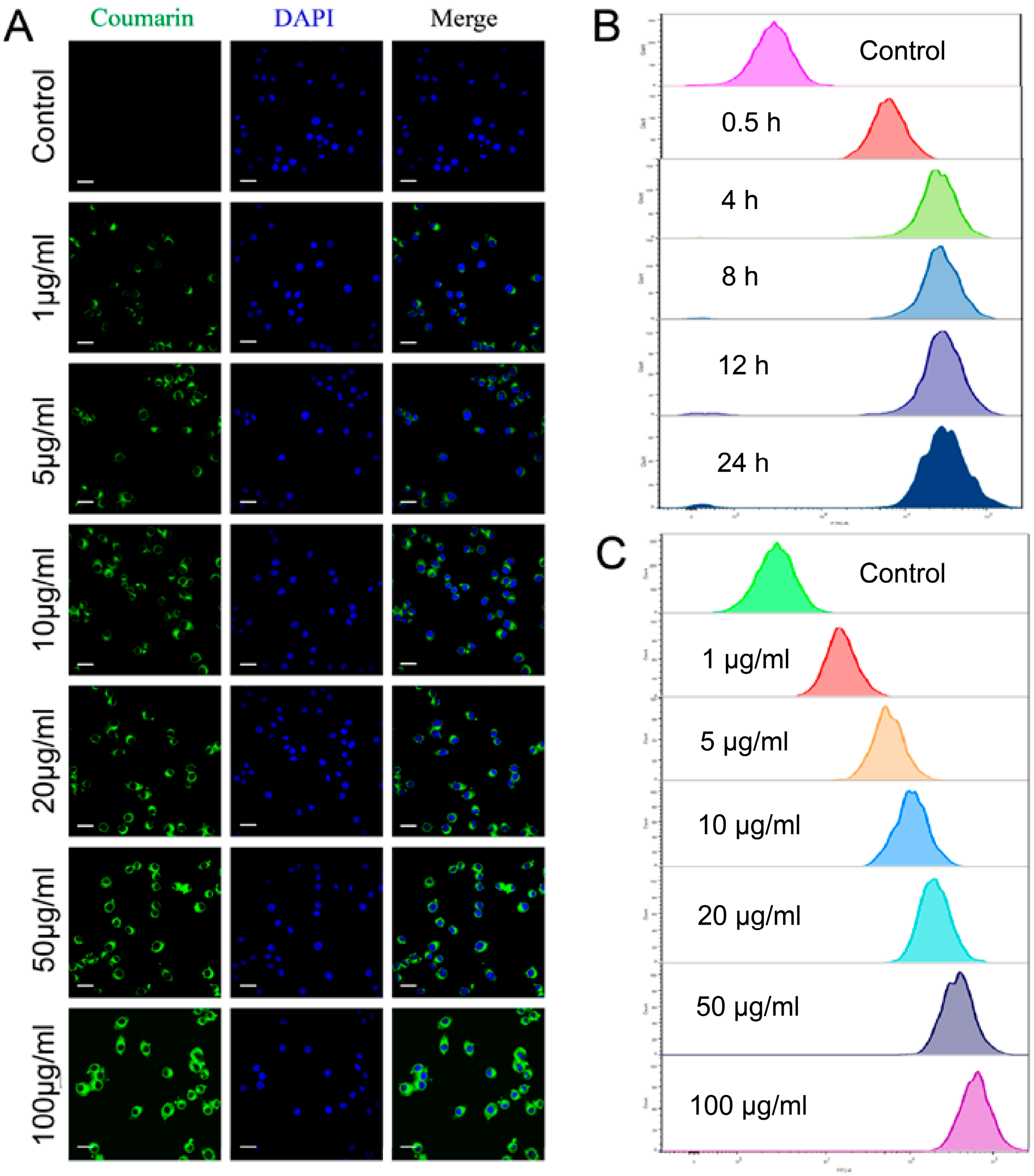

Figure S9. Confocal microscopy images of the concentration-dependent cellular uptake of mAb-C6@HCNPs. After activated RAW264.7 cells were incubated with mAb-C6@HCNPs (green) at various concentrations (A), nuclei were stained with DAPI (blue). Scale bar: 20 μm. The corresponding quantitative analysis of time-dependent cellular uptake for mAb-C6@HCNPs by flow cytometry (B). Quantitative analysis of the concentration-dependent cellular uptake of mAb-C6@HCNPs by flow cytometry is also shown (C).

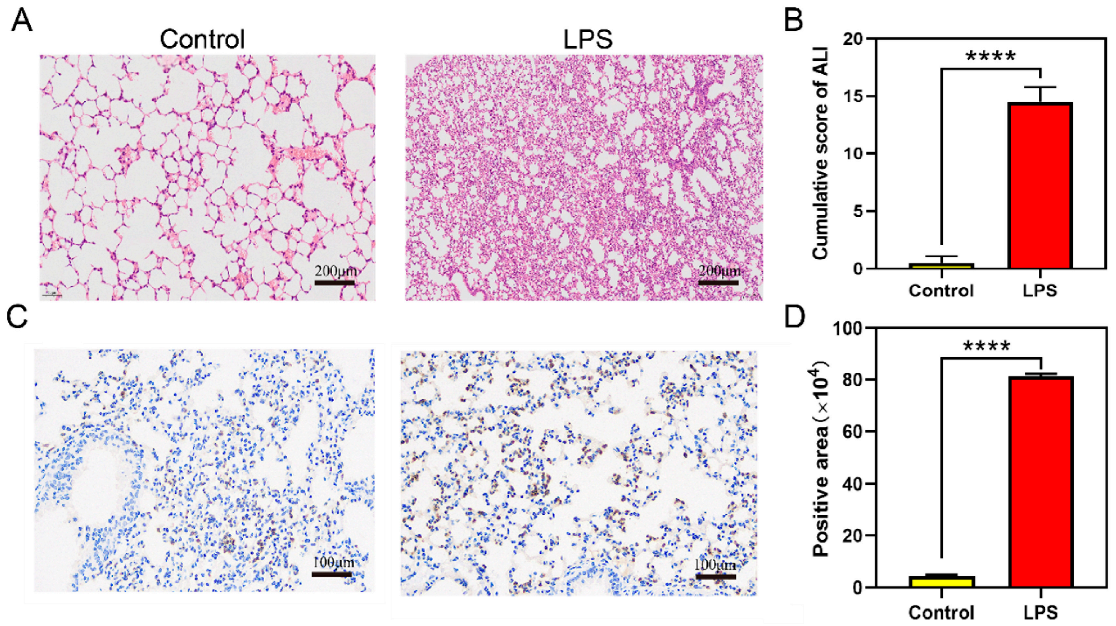

Figure S10. Establishment and validation of the mouse sepsis ALI model. Hematoxylin and eosin (H&E)-stained images of lung sections (A) and the cumulative ALI score are also shown (B). The scale bar is 200 μm. MPO immunohistochemistry (IHC) of lung sections (C) and corresponding quantification results (D); the scale bar is 100 μm.

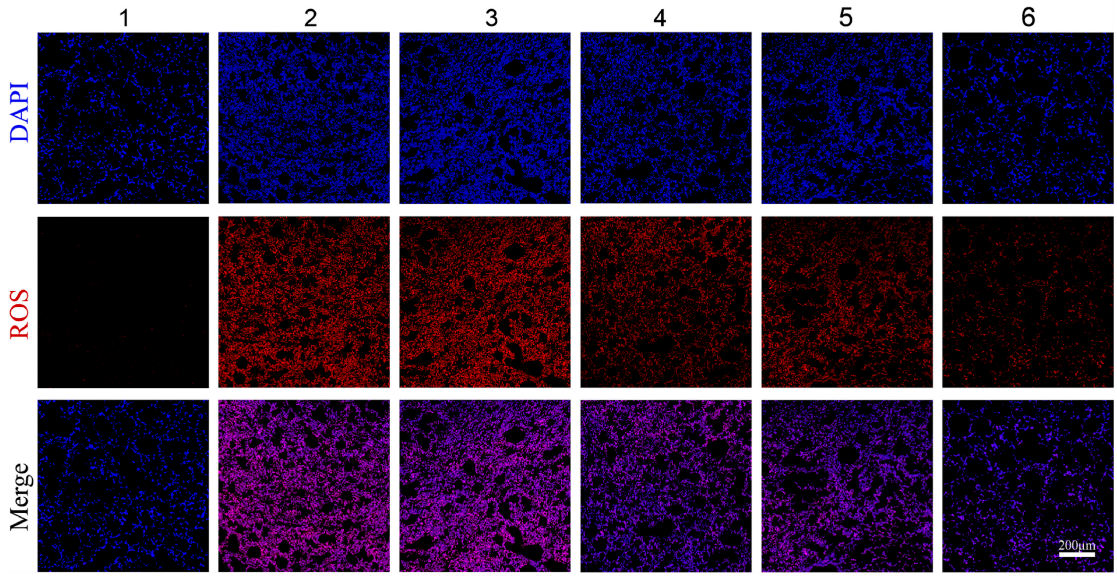

Figure S11. Representative fluorescence images visualizing ROS. DHE and DAPI staining of lung tissues (blue, nuclei; red, ROS). Groups 1-6 represent the control, LPS, LPS+HCNPs, LPS+TPCA-1, LPS+IgG-TPCA-1@HCNPs and LPS+mAb-TPCA-1@HCNPs groups respectively. Scale bars, 200 μm.

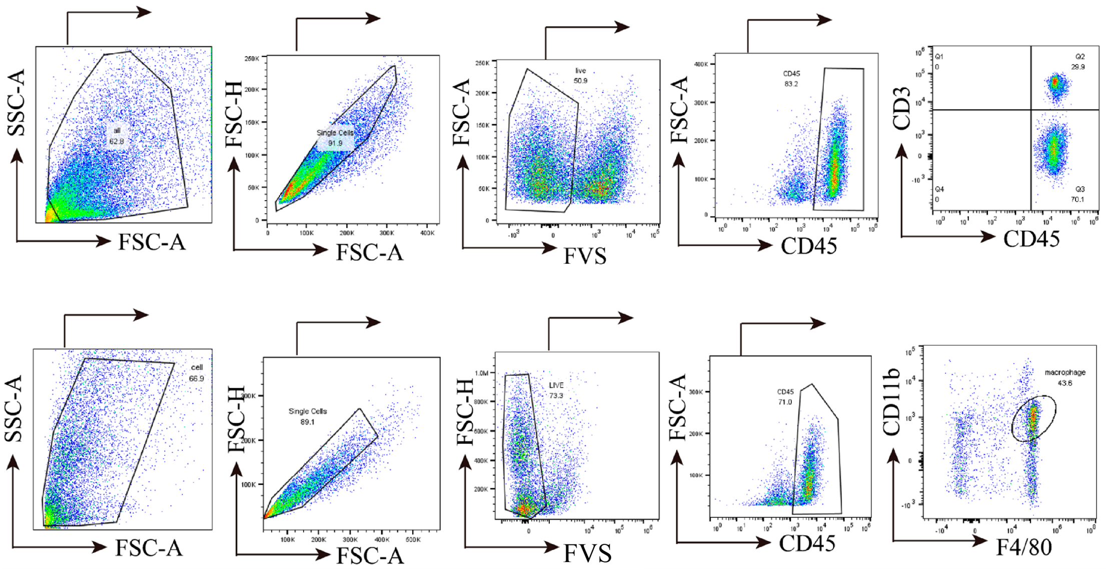

Figure S12. Gating strategy for flow cytometry analysis used to detect T cells (upper) and macrophages (lower).

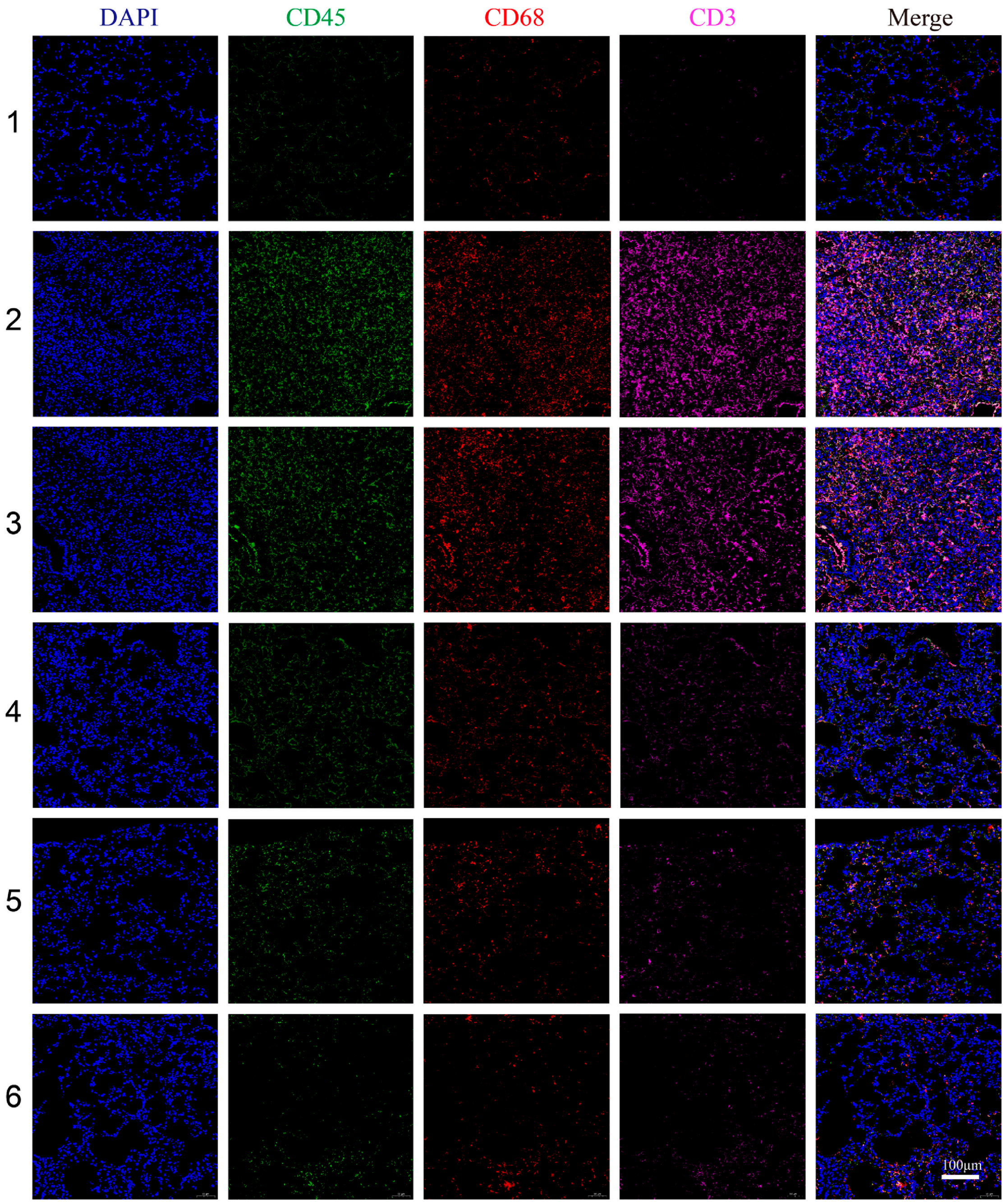

Figure S13. Representative immunofluorescence images of lung tissue sections in different groups as indicated (blue, nuclei; green, CD45; red, CD68; pink, CD3). Groups 1~6 represent the control, LPS, LPS+HCNPs, LPS+TPCA-1, LPS+IgG-TPCA-1@HCNPs and LPS+mAb-TPCA-1@HCNPs groups. Scale bars, 100 μm.

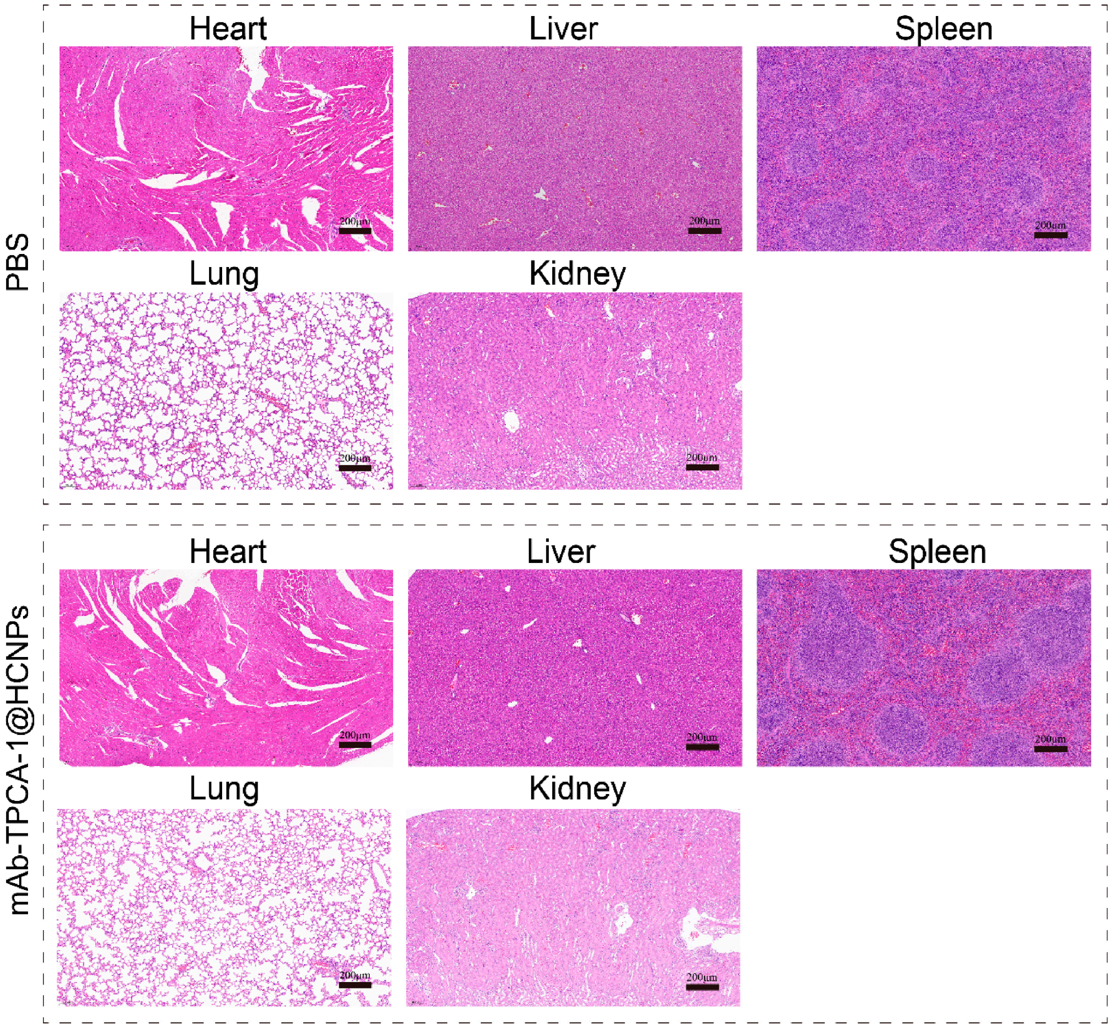

Figure S14. H&E staining of the heart, liver, spleen, lung, and kidney after 14 days of treatment with PBS and mAb-TPCA-1@HCNPs at a high dose of 100 mg/kg in healthy BALB/c mice. Scale bar is 200 μm.
